# Successful Haplo‐Hematopoietic Stem Cell Transplantation for Juvenile Myelomonocytic Leukemia in a Child With Underlying Thrombocytopenia‐Absent Radius Syndrome: A Unique Case

**DOI:** 10.1002/cnr2.70523

**Published:** 2026-04-02

**Authors:** Sondus Al Sharidah, Ahmed Elhussien, Walid I. A. Soliman, Nesma I. Ellithy

**Affiliations:** ^1^ Pediatric Hematology/Oncology and Stem Cell Transplant Unit NBK Specialized Hospital for Children Kuwait

**Keywords:** congenital thrombocytopenia, hematopoietic stem cell transplantation, JMML, juvenile myelomonocytic leukemia, NF1 mutation, pediatric leukemia, TAR syndrome

## Abstract

**Background:**

Thrombocytopenia‐absent radius (TAR) syndrome is a rare congenital disorder characterized by bilateral radial aplasia with preserved thumbs and early‐onset thrombocytopenia. While hematologic and skeletal abnormalities define the condition, its association with hematologic malignancies is extremely rare, with only a few reported cases of leukemia. Juvenile myelomonocytic leukemia (JMML) is an uncommon pediatric myelodysplastic/myeloproliferative neoplasm frequently linked to RAS pathway mutations. To our knowledge, JMML has not previously been reported in association with TAR syndrome.

**Case Presentation:**

We report the case of a male infant diagnosed with TAR syndrome based on clinical features and molecular confirmation of a homozygous *RBM8A* c.‐21G>A variant. The patient presented initially with persistent thrombocytopenia, skeletal deformities, and neonatal sepsis‐like manifestations. At 2 years of age, he developed pancytopenia and progressive splenomegaly. Bone marrow evaluation and molecular testing confirmed JMML harboring a pathogenic *NF1* mutation. He underwent successful haploidentical hematopoietic stem cell transplantation (HSCT) from a sibling donor, following a conditioning regimen of melphalan, treosulfan, cyclophosphamide, and anti‐thymocyte globulin. The patient achieved full donor chimerism and hematologic remission with stable engraftment.

**Conclusion:**

This case represents, to our knowledge, one of the very few—if not the first—reported instances of successful HSCT for JMML in a patient with TAR syndrome. It underscores the importance of vigilant surveillance in TAR patients for potential malignant transformation and demonstrates the curative potential of HSCT in rare congenital‐hematologic overlap syndromes.

## 1 Introduction

Thrombocytopenia‐absent radius (TAR) syndrome is a rare congenital disorder characterized by bilateral radial aplasia with preserved thumbs and early‐onset thrombocytopenia, typically developing within the first weeks of life, with platelet counts often below 50 × 10^9^/L. Its worldwide prevalence is estimated at ~1 in 100 000–200 000 live births [1, 2, 3]. Although TAR syndrome is primarily recognized for its hematological and skeletal manifestations, its association with leukemia is extremely uncommon. To date, only a few cases of leukemia have been documented in patients with TAR syndrome: four cases of acute myeloid leukemia (AML)—two in children [4, 5] and two in adults aged 42 and 47 years [1, 2]—and one pediatric case of acute lymphoblastic leukemia (ALL) [6].

Mortality in TAR syndrome, mainly due to hemorrhage, is generally confined to the first year of life, after which survivors usually have a normal life expectancy [1]. However, children with TAR who develop leukemia have historically experienced poor outcomes, either fatal [4, 5] or with relapse after remission [6].

Here, we present an exceptional case of juvenile myelomonocytic leukemia (JMML) occurring in association with TAR syndrome.

## 2 Case Report

A 33‐day‐old male infant was referred to our hospital with a provisional diagnosis of TAR syndrome for further evaluation. Born in 2022, he was admitted to the NICU for respiratory distress, with chest X‐ray findings suggestive of transient tachypnea of the newborn. He was also diagnosed with neonatal sepsis, persistent thrombocytopenia, and leukocytosis. Empirical antibiotic therapy was initiated; however, blood cultures were negative. Over the following weeks, his clinical condition remained poor yet stable.

**FIGURE 1:**
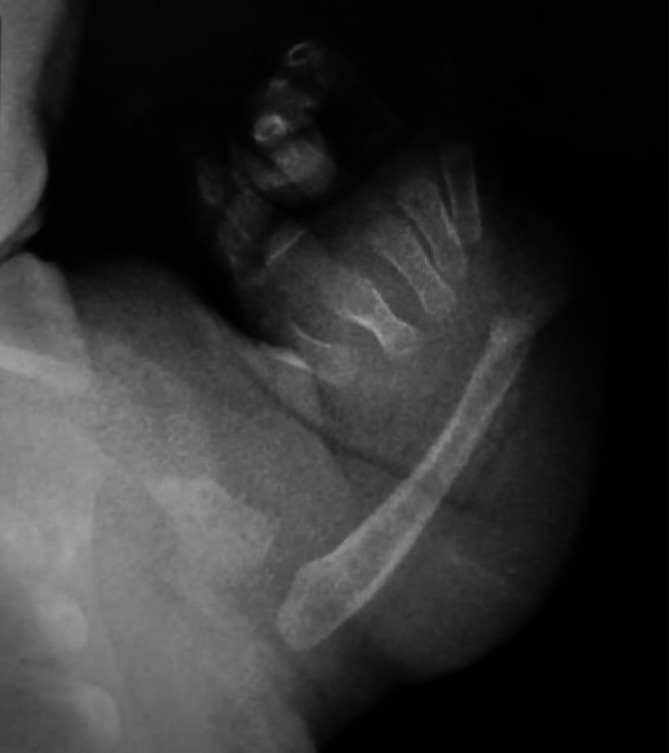
Anteroposterior X‐ray of the forearm demonstrating characteristic bilateral absence of the radii with preserved thumbs, consistent with TAR syndrome.

At presentation to our clinic, the patient was clinically stable with vital signs within normal limits. Physical examination revealed bilateral limb deformities and mild splenomegaly. Laboratory analysis showed a platelet count of 35 × 10^9/L (thrombocytopenia), which was managed with platelet transfusion. A limb X‐ray confirmed the bilateral absence of the radii with preserved thumbs (Figure 1). Based on these findings, a molecular genetic panel was ordered. Next‐generation sequencing (NGS) revealed a homozygous c.‐21G>A mutation in RBM8A, consistent with TAR syndrome. Parental testing confirmed both parents were heterozygous carriers of the RBM8A mutation. NGS testing for the *NF1* variant (c.2915T>C; p.L972P) was performed on both bone marrow and peripheral blood samples, consistent with JMML but without clinical features of neurofibromatosis. This finding is interpreted as a somatic mutation in the context of JMML. In contrast, the *RBM8A* c.‐21G>A variant was identified in peripheral blood and confirmed as germline through parental testing, which demonstrated both parents were heterozygous carriers.

A bone marrow aspirate was performed to evaluate for malignancy, which showed 1% blasts—below the diagnostic threshold for leukemia. Family history revealed a non‐consanguineous marriage with no history of malignancy on either side, reducing the likelihood of a hereditary predisposition to leukemia.

The patient continued regular follow‐ups, frequently presenting with melena. Investigations confirmed hematochezia and persistent thrombocytopenia, necessitating ongoing platelet transfusions to maintain platelet counts of 7–10 ×10^9^/L. In 2024, at the age of 2 years, he developed pancytopenia and progressive splenomegaly. A peripheral blood smear revealed 8% blasts. Molecular studies identified a pathogenic **NF1** gene mutation (c.2915 T>C, p.L972P) in the absence of clinical features of neurofibromatosis. These findings were diagnostic of **juvenile myelomonocytic leukemia (JMML)**.

Bone marrow biopsy revealed active myelopoiesis with an increased eosinophilic lineage, moderate erythropoiesis, absent megakaryopoiesis, and 7% blasts. Immunophenotyping indicated 5% myeloblasts expressing CD33, CD34, CD117, and HLA‐DR.

According to the WHO Classification of Haematolymphoid Tumours (5th edition) [7], the patient fulfilled the Genetic Criteria (Category 2) for JMML due to the presence of a somatic NF1 mutation. Coupled with the bilateral absence of radii and thrombocytopenia, a diagnosis of JMML associated with TAR syndrome was confirmed.

Hematopoietic stem cell transplantation (HSCT) was pursued. The patient's sibling (brother) was identified as a suitable haploidentical donor with a 9/10 HLA match. The CD34^+^ cell dose was 7.2 × 10^6 cells/kg. The pre‐transplant conditioning regimen consisted of treosulfan 14 g/m^2^/day on days −7, −6, and − 5 (total 42 g/m^2^), cyclophosphamide 60 mg/kg on day −4, melphalan 140 mg/m^2^ on day −2, and rabbit anti‐thymocyte globulin (rATG) 2.5 mg/kg/day on days −3 and − 2 (total 5 mg/kg). Graft‐versus‐host disease (GVHD) prophylaxis included post‐transplant cyclophosphamide (PTCy) 50 mg/kg on days +3 and + 4 with Mesna uroprotection, followed by mycophenolate mofetil (MMF) and sirolimus.

On day 13 post‐transplant, the patient developed an acute skin rash consistent with engraftment syndrome in the absence of infection. Neutrophil engraftment occurred on day +20, and platelet engraftment on day +37 post‐transplant. The platelet count subsequently increased to 224 × 10^9^/L. Donor chimerism analysis demonstrated full donor engraftment in lymphoid, myeloid, and whole blood compartments, as shown in Table 1.

**TABLE 1:** Post‐transplant donor chimerism in patient.

Sample type	Day + 28 Mean donor chimerism (%)	Day + 90 Mean donor chimerism (%)
Lymphocyte fraction (lymphoid chimera)	96.61	98.84
Myelocyte fraction (myeloid chimera)	97.97	99.53
Whole Blood	96.50	100

The patient's post‐transplant course was favorable. Frequent follow‐ups and weekly monitoring demonstrated stable complete blood counts. A recent bone marrow aspirate performed on day +30 & day +60 showed a normocellular marrow with < 5% blasts, confirming continued remission and normal hematopoietic function. At the most recent follow‐up (+17 months post‐transplant), the patient remains in complete remission and is clinically active and well with no complaints. He is fully engrafted with sustained donor chimerism and has stable hematologic recovery with satisfactory CBC. The patient has commenced revaccination according to international post‐HSCT guidelines and continues with regular physiotherapy and rehabilitation follow‐up, maintaining good functional status and daily activity.

## 3 Discussion

TAR syndrome is caused by pathogenic variants in the ***RBM8A*** gene, which encodes RNA‐binding motif protein 8A (Y14). The identification of the ***RBM8A* c.‐21G>A** variant, along with the clinical features observed in this case, confirmed the diagnosis [3, 8].

TAR syndrome typically results from compound heterozygosity involving a null allele (most often a 1q21.1 deletion of RBM8A) and a hypomorphic allele, and follows an autosomal recessive inheritance pattern. Parents of affected individuals are usually heterozygous carriers and asymptomatic [3]. Therefore, genetic testing of both the patient and parents is essential for definitive diagnosis and clarification of the inheritance pattern.

Lesions in **RBM8A**—including microdeletion at 1q21.1 and single nucleotide polymorphisms in coding or non‐coding regions—result in reduced **RBM8A** expression. This leads to deficiency of the Y14 subunit of exon‐junction complex, a protein essential for RNA processing and regulation [8]. Disruption of this pathway impairs the development of multiple tissues, particularly hematopoietic and skeletal systems [9, 10].

Albers et al. suggested that complex interactions between RNA‐binding proteins, including the translational repressor Evi‐1, and aberrant RBM8A binding sites may underlie the skeletal deformities in TAR syndrome [10]. In rodent models, Evi‐1 has been shown to be transiently expressed in developing limb buds [11].

The thrombocytopenia characteristic of TAR syndrome is thought to result from defective thrombopoietin–c‐Mpl receptor signaling, impairing megakaryocyte differentiation [8].

Only a few pediatric cases of leukemia associated with TAR syndrome have been reported. In 1993, Rock and Camitta described a 5‐year‐old girl with TAR syndrome and acute lymphoblastic leukemia who achieved remission with modified chemotherapy, relapsed 2 years later, and subsequently attained a second remission [6]. Fadoo et al. reported a 1‐year‐old boy with TAR syndrome and acute myeloid leukemia (AML, M2), who died within 1 month of diagnosis before receiving chemotherapy [5]. Rao et al. described a fatal case of AML in a 2‐month‐old infant with TAR syndrome [4]. Collectively, these reports suggest that patients with TAR may have a predisposition to leukemia, often with poor outcomes.

JMML is a rare, aggressive myelodysplastic/myeloproliferative disorder that typically occurs in infancy or early childhood. It is characterized by anemia, thrombocytopenia, leukocytosis, and monocytosis, and is driven by hypersensitive myeloid progenitors [12]. JMML is frequently associated with somatic or germline mutations in RAS pathway genes, including *NF1*, *PTPN11*, *KRAS*, *NRAS*, and *CBL* [7, 13, 14].

Clinical manifestations of JMML—such as respiratory distress, hepatosplenomegaly, pallor, fever, and rashes—can mimic neonatal sepsis, particularly in early‐onset cases [13]. In our patient, JMML initially presented as sepsis and respiratory distress but was later confirmed through molecular identification of an *NF1* mutation, bone marrow examination, and immunophenotyping. Given this overlap, infections must be excluded before establishing a diagnosis of JMML [13].

The association between TAR syndrome and leukemia may reflect shared or intersecting genetic mechanisms. Jameson‐Lee et al. in their case report and review proposed that downregulation of Y14, a spliceosome complex component, may disrupt RNA processing in a manner that promotes leukemogenesis [1]. Since spliceosome‐related mutations are implicated in de novo AML, impaired ***RBM8A*** function could contribute to leukemia transformation in TAR syndrome.

Two adult cases of TAR‐associated leukemia have also been reported. Go et al. described a 42‐year‐old woman with TAR syndrome and AML who achieved remission after intensive chemotherapy [2], a treatment not feasible in infants. Jameson‐Lee et al. described a 47‐year‐old man with TAR syndrome and myelodysplastic syndrome progressing to AML, successfully treated with hematopoietic stem cell transplantation (HSCT) [1]. These cases suggest that HSCT may represent a potentially curative option in TAR‐associated hematologic malignancies, although evidence remains limited due to the very small number of reported patients.

Conventional chemotherapy is generally ineffective in JMML, with mortality exceeding 90% due to resistance and treatment‐related toxicity in young children. Recent larger cohorts report 5‐year overall survival after HSCT in JMML of approximately 70%–75%, reflecting improvements in transplant techniques, risk stratification, and supportive care [15, 16]. Our patient underwent haploidentical HSCT from a sibling donor and achieved full donor chimerism, normalized blood counts, and resolution of clinical symptoms. To our knowledge, this represents one of the very few—if not the first—documented cases of successful HSCT for JMML in the context of TAR syndrome.

## 4 Conclusion

The rare co‐occurrence of TAR syndrome and JMML, together with increasing reports of leukemia in TAR patients, suggests a potentially underrecognized predisposition to hematologic malignancies in this population. Given the diagnostic challenges and historically poor outcomes associated with this dual pathology, vigilant clinical surveillance and early molecular evaluation are essential. In particular, clinicians should closely monitor TAR patients for persistent or worsening cytopenias, progressive splenomegaly, and recurrent sepsis‐like presentations, as these features may indicate leukemic transformation. Recognition of such clinical red flags should prompt early referral for confirmatory molecular testing, especially targeting RAS pathway mutations, to establish a timely and accurate diagnosis.

Furthermore, this case emphasizes the feasibility and therapeutic potential of hematopoietic stem cell transplantation (HSCT) in achieving durable remission, even in the setting of complex congenital syndromes. To our knowledge, this is one of the very few—if not the first—reported cases of successful HSCT for JMML in a patient with TAR syndrome, underscoring the need for further research into shared pathogenic mechanisms and long‐term treatment outcomes.

## Author Contributions

**Sondus Al Sharidah:** conceptualization, investigation, writing – review and editing, formal analysis, supervision. **Ahmed Elhussien:** designed, methodology, writing – original draft. **Walid I. A. Soliman:** data curation. **Nesma I. Ellithy:** data curation. All authors approved the final version of this article for the submission.

## Funding

The publication of this article is supported by National Bank of Kuwait (NBK) as part of its Corporate Social Responsibility Program.

## Consent

Written informed consent was obtained from legal guardians for the publication of any potentially identifiable images or data included in this article.

## Conflicts of Interest

The authors declare no conflicts of interest.

## Data Availability

The data that support the findings of this study are available on request from the corresponding author. The data are not publicly available due to privacy or ethical restrictions.
